# Illustration of self-perceived knowledge, skills, and interests in undergraduate dental students using a visual metaphor– results of a monocentric cross-sectional study

**DOI:** 10.1186/s12909-024-05257-w

**Published:** 2024-03-12

**Authors:** Gerhard Schmalz, Stefan Büchi, Rainer Haak, Dirk Ziebolz, Maria Strauß

**Affiliations:** 1 Department of Cariology, Endodontology and Periodontology, University of Leipzig, Liebigstr. 12, 04103 Leipzig, Germany; 2 mediX Gruppenpraxis Rotbuchstrasse, Zürich, Switzerland; 3 Department of Psychiatry and Psychotherapy, University of Leipzig, Leipzig, Germany

**Keywords:** Dental education, PRISM, Visual metaphor, Undergraduate education, Self-reflection, Communication

## Abstract

**Background:**

Self-assessment and self-reflection of competencies are crucial skills for undergraduate students. This monocentric cross-sectional study aims to assess the self-perceived knowledge, skills and interests in conservative dentistry and periodontology of third-, fourth-, and fifth-year dental students by the Pictorial Representation of Illness and Self-measure (PRISM).

**Methods:**

Seventy-five undergraduate dental students (*n* = 25 of each year) who studied between 2021 and 2022 at the Department of Cariology, Endodontology and Periodontology at the University of Leipzig, Germany, were included. All of them underwent a PRISM-based interview regarding their perceived knowledge, practical skills, and interests in conservative dentistry as well as its sub-disciplines. The distances in the PRISM task (in millimeters) were measured and compared between the groups. Spearman’s Rho was used to reveal correlations between knowledge, skills, and interests in the cohort.

**Results:**

Perceived theoretical knowledge and practical skills differed significantly between groups for the sub-disciplines periodontology, cariology, restorative dentistry and preventive dentistry (*p* < 0.05). However, students’ interests did not significantly vary between groups (*p* > 0.05). In the field of conservative dentistry and its sub-disciplines, significant moderate to high positive correlations were found between knowledge and skills (*p* < 0.01), and weak to moderate positive correlations were found between interests and knowledge (*p* < 0.05). Regarding the relationship between perceived interests and skills, only restorative dentistry, endodontology and periodontology were significant and only moderate to weak correlations were found (*p* < 0.05).

**Conclusion:**

PRISM revealed differences in perceived knowledge and skills between third-, fourth-, and fifth-year dental students. Correlations were found between perceived knowledge and skills, as well as between interests and knowledge. PRISM may be a promising tool to support students and teachers in dental education.

## Background

Assessing dental students’ experience, self-perceived competencies, and confidence with different treatment procedures is important in dental education and this topic is drawing increasing interest in the field [1–4]. Therefore, self-perceived issues are regularly assessed using numerical or rubric questionnaires or visual analogue scales [1–5].

Recently, a novel instrument that can visualize self-perceived knowledge, skills, interest, and learning needs in an alternative way was introduced. The Pictorial Representation of Illness and Self Measure (PRISM) is a visual metaphor that originates from the field of psychosomatics [6]. The basic principle relies on the placement of different objects to fixed subjects to represent the relationships between them. In medicine, this method facilitates the evaluation of personally salient information from the patient [7]. In the context of dental education, PRISM was used to depict self-perceived knowledge and skills in dental students. This evaluation was found to be reproducible and sensitive to changes in teaching [8]. Furthermore, PRISM was found to be superior to a numeric scale from the perspective of clinical undergraduate dental students [9]. Therefore, using PRISM as an alternative evaluation could support self-reflection and personal academic achievement alongside learning progress in dental students. This would support the acquisition of important skills for dental and general medical education, including a lifelong learning process [10], a trustful relationship between students and teachers [11], and self-reflection as a primary skill in dental education [12]. Therefore, PRISM in dental education appears to be a promising approach for dental research, especially in the context of self-perceived knowledge, skills and academic interests.

Until now, much information is still missing in the usage of PRISM. One issue is whether it would be possible to depict differences in PRISM-based evaluation of knowledge and skills between students in different years of study. This would provide a deeper understanding of the informative value of PRISM in this context and on the differences in self-perception between students at different levels of progress. Additionally, whether there are correlations between perceived knowledge, skills, and interests revealed by the PRISM interviews is also of interest. Previous studies have shown heterogeneous correlations between theoretical knowledge and practical skills using different approaches to compare knowledge and practical competencies [13–15]. Therefore, the students’ perspective appears to be meaningful in this context.

Overall, the current study had two aims. The first aim is to use the PRISM task to reveal the self-perceived knowledge, skills, and interests in conservative dentistry and periodontology of third-, fourth-, and fifth-year dental students and make comparisons between the three groups. The second aim is to evaluate whether the perceived skills, knowledge, and interests in the broader cohort are correlated with each other. It was hypothesized that: (I) perceived knowledge and skills increase with greater years of dental studies, and (II) knowledge, skills, and interests are correlated with each other. Therefore, the respective null hypotheses were that there would be no differences in perceived knowledge and skills across years of study and that there would be no correlations between knowledge, skills, and interest.

## Methods

### Study design

This monocentric cross-sectional cohort study included students from three different years of undergraduate dental study. The ethics committee of the medical faculty of the University of Leipzig, Germany reviewed and approved the protocol of the current study (No: 117/20-ek). All participating students were volunteers who gave their written informed consent for participation.

### Setting, participants and groups

A cohort of clinical dental students who studied between 2021 and 2022 at the Department of Cariology, Endodontology and Periodontology, University of Leipzig, Germany, was recruited. There were three subgroups of third-, fourth-, and fifth-year students.

This study aimed to compare the different subgroups to each other. For this purpose, sample size calculation was applied based on previous studies in the field [8, 9]. To reach a power of at least 0.8, considering a type error rate of 5%, a sample size of at least 22 per group is required. Therefore, for each year of study, a randomly selected sample of 25 volunteers was chosen. First, all students of each year were contacted via email within their recent (pre-)clinical courses, informed about the study, and asked to voluntarily participate. Altogether, 32 third-year students, 35 fourth-year students, and 36 fifth-year students indicated their interest in participation (the typical size of a cohort is 46 students). The mandatory inclusion criteria were being in the third, fourth, or fifth year of dental studies, and voluntary participation. The only exclusion criterion was current participation in another PRISM-based study. There were no additional inclusion or exclusion criteria. All of the interested students fulfilled those criteria. Out of the respective groups of interested students, 25 participants per study year were randomly selected by the drawing of lots and included after written informed consent for participation.

### Principle of PRISM

**Fig. 1:**
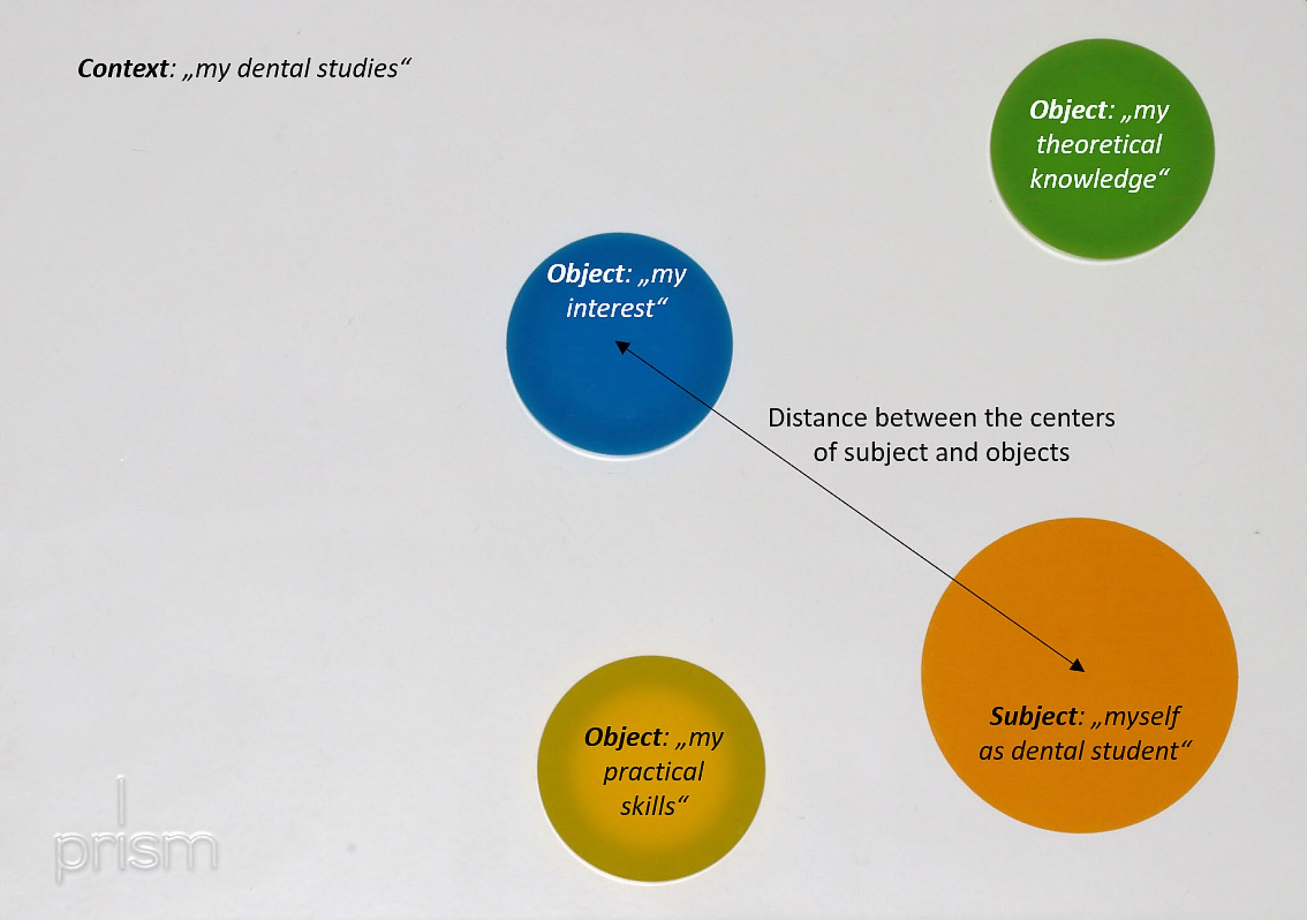
Principle of PRISM used in the current study. A subject disc reflects the students’ self, while the object discs included skills, knowledge, and interests in the disciplines of conservative dentistry and periodontology. The results of the current study were the distances between the centers of the respective discs

The approach of using PRISM in dental education has been explained previously in detail [8]. In brief, the method originates from psychology/psychosomatic medicine and is performed based on a white metal board (297 × 210 mm; “context”) with a fixed yellow subject circle (7 cm in diameter) at the bottom right-hand corner (“subject”). Additionally, different object discs (5 cm diameter) can be placed in relation to the yellow circle. For the current study, perceived “theoretical knowledge,” “practical skills,” and “interest” were chosen as the object discs. Objects for the whole field of conservative dentistry and periodontology, as well as for its five sub-disciplines, namely endodontology, periodontology, cariology, restorative dentistry, and preventive dentistry, were placed. The distances between the centers of the object and subject discs, which are the outcome parameters, were measured in millimeters, as depicted in Fig. 1.

This distance represents the relative importance of the individual object, whereby the closer an object is to the subject, the more relevant it is for the student.

### PRISM interviews

A trained and experienced dentist (GS) performed all PRISM interviews under comparable conditions (room and setting). The general process of a PRISM interview in dental education has been described in previous studies [8, 9]. In brief, the interview started with an explanation of the PRISM task, followed by one example question. Within the interviews, students had to place the objects, which reflected their perceived knowledge, skills, and interests for each of the sub-disciplines as well as the overall field of conservative dentistry. As mentioned above, the closer the object was placed to the subject, the greater students perceived their skills or the higher their interest. As performed previously, the distance between the subject disc and object discs was measured in millimeters using a millimeter-scaled ruler [8]. The distances in millimeters were recorded for each subject-object relationship for knowledge, skills, and interest. Those distances were used for analysis of comparisons between study years and to reveal potential correlations. The time for the interviews ranged between eight and ten minutes for each student. All of the students were only interviewed once.

### Statistical analysis

The statistical analysis was performed with SPSS for Windows, version 24.0 (SPSS Inc., U.S.A.). First, a normal distribution was checked by the Kolmogorov-Smirnov test, which indicated non-normal distribution for nearly all of the tested variables (*p* < 0.05). Thus, nonparametric tests for nonnormal distributed samples were chosen. Accordingly, more than two independent, non-normally distributed samples were compared by the Kruskal-Wallis test. Categorical or nominal data were analyzed by the chi-square test. The correlations between perceived knowledge and skills, interest, and skills and interest and knowledge were evaluated using Spearman’s Rho. Regarding the correlation coefficient, the following interpretations were made:

0.1–0.39 = weak correlation, 0.4–0.69 = moderate correlation, 0.7–0.89 = strong correlation and 0.9-1 = very strong correlation [16]. Two-sided significance testing was performed for all of the values, whereby the significance level was set at *p* < 0.05.

## Results

### Participants

Seventy-five students, with a mean age of 23.8 ± 2.8 years (37.3% male gender), were included in the study. Therefore, 25 third-year students (23.0 ± 3.2 years, 28% male), 25 fourth-year students (23.1 ± 2.3, 36% male) and 25 fifth–year students (25.4 ± 2.8, 48% male) could be evaluated based on their respective groups. Thereby, age differed significantly between groups (*p* < 0.01), while gender did not (*p* = 0.34).

### Knowledge, skills, and interest between different study years

Regarding perceived theoretical knowledge, differences were found between groups for the sub-disciplines periodontology, cariology, restorative dentistry, and preventive dentistry (*p* < 0.05). In post-hoc analysis, these differences were primarily confirmed between third- and fifth-year students in periodontology, restorative and preventive dentistry (*p* < 0.01). The detailed results can be seen in Table 1.

**Table 1:** Results of the perception of individual theoretical knowledge between groups. Data are presented as median (IQR)

	3rd year students (*n* = 25)	4th year students (*n* = 25)	5th year students (*n* = 25)	*p* value
Whole field of conservative dentistry and periodontology	54 (26–82)	42 (34–54)	30 (17–45)	0.06
Endodontology	60 (30–84)	56 (28–74)	27 (10–63)	0.11
Periodontology	60 (30–97)^a^	43 (20–75)^b^	11 (3–30)^ab^	< 0.01
Cariology	60 (34–106)	28 (12–56)	30 (15–56)	0.04
Restorative dentistry	87 (60–110)^a^	81 (36–92)^b^	30 (15–57)^ab^	< 0.01
Preventive dentistry	77 (28–115)^ac^	16 (3–28)^c^	10 (2–26)^a^	< 0.01

Values are given as the distance between the object and subject disc in millimeters, shown as the mean values and standard deviation

^a^Significant difference in post-hoc testing between the 3rd and 5th years (*p* ≤ 0.01)

^b^Significant difference in post-hoc testing between the 4th and 5th years (*p* ≤ 0.01)

^c^Significant difference in post-hoc testing between the 3rd and 4th years (*p* ≤ 0.01)

In the perceived skills of the students, differences were found between the groups for the overall field of conservative dentistry, as well as for the sub-disciplines periodontology, cariology, and restorative and preventive dentistry (*p* < 0.01). The post-hoc analysis revealed significant differences between third- and fifth-year students as well as between third- and fourth-year students for all sub-disciplines (*p* < 0.01, Table 2).

**Table 2:** Results of the perception of individual practical skills between groups. Data are presented as median (IQR)

	3rd year students (*n* = 25)	4th year students (*n* = 25)	5th year students (*n* = 25)	*p* value
Whole field of conservative dentistry and periodontology	72 (43–100)^a^	52 (30–58)	29 (10–52)^a^	< 0.01
Endodontology	76 (37–100)	80 (58–134)	55 (33–100)	0.23
Periodontology	135 (72–186)^ac^	47 (33–70)^bc^	12 (0–30)^ab^	< 0.01
Cariology	80 (46–115)^ac^	32 (14–63)^c^	20 (9–40)^a^	< 0.01
Restorative dentistry	100 (68–117)^ac^	72 (33–87)^c^	33 (10–82)^a^	< 0.01
Preventive dentistry	104 (151 − 72)^ac^	13 (3–25)^c^	6 (0–15)^a^	< 0.01

Values are given as the distance between the object and subject disc in millimeters, shown as the mean values and standard deviation

^a^Significant difference in post-hoc testing between the 3rd and 5th years (*p* ≤ 0.01)

^b^Significant difference in post-hoc testing between the 4th and 5th years (*p* ≤ 0.01)

^c^Significant difference in post-hoc testing between the 3rd and 4th years (*p* ≤ 0.01)

No significant differences in interests were confirmed between the groups (*p* > 0.05, Table 3). Overall, the distances were comparably small for all groups, indicating high interest in all groups.

**Table 3:** Results of the perception of individual interests between groups. Data are presented as median (IQR)

	3rd year students (*n* = 25)	4th year students (*n* = 25)	5th year students (*n* = 25)	*p* value
Whole field of conservative dentistry and periodontology	0 (0–16)	13 (0–22)	5 (0–30)	0.28
Endodontology	12 (0–42)	18 (0–28)	32 (3–70)	0.19
Periodontology	14 (0–42)	20 (10–38)	10 (0–24)	0.17
Cariology	5 (0–40)	29 (3–44)	21 (6–40)	0.46
Restorative dentistry	40 (12–65)	25 (11–37)	26 (12–37)	0.52
Preventive dentistry	0 (0–20)	12 (0–45)	5 (0–38)	0.57

Values are given as the distance between the object and subject disc in millimeters, shown as the mean values and standard deviation

*Correlations between perceived knowledge and skills*.

**Fig. 2:**
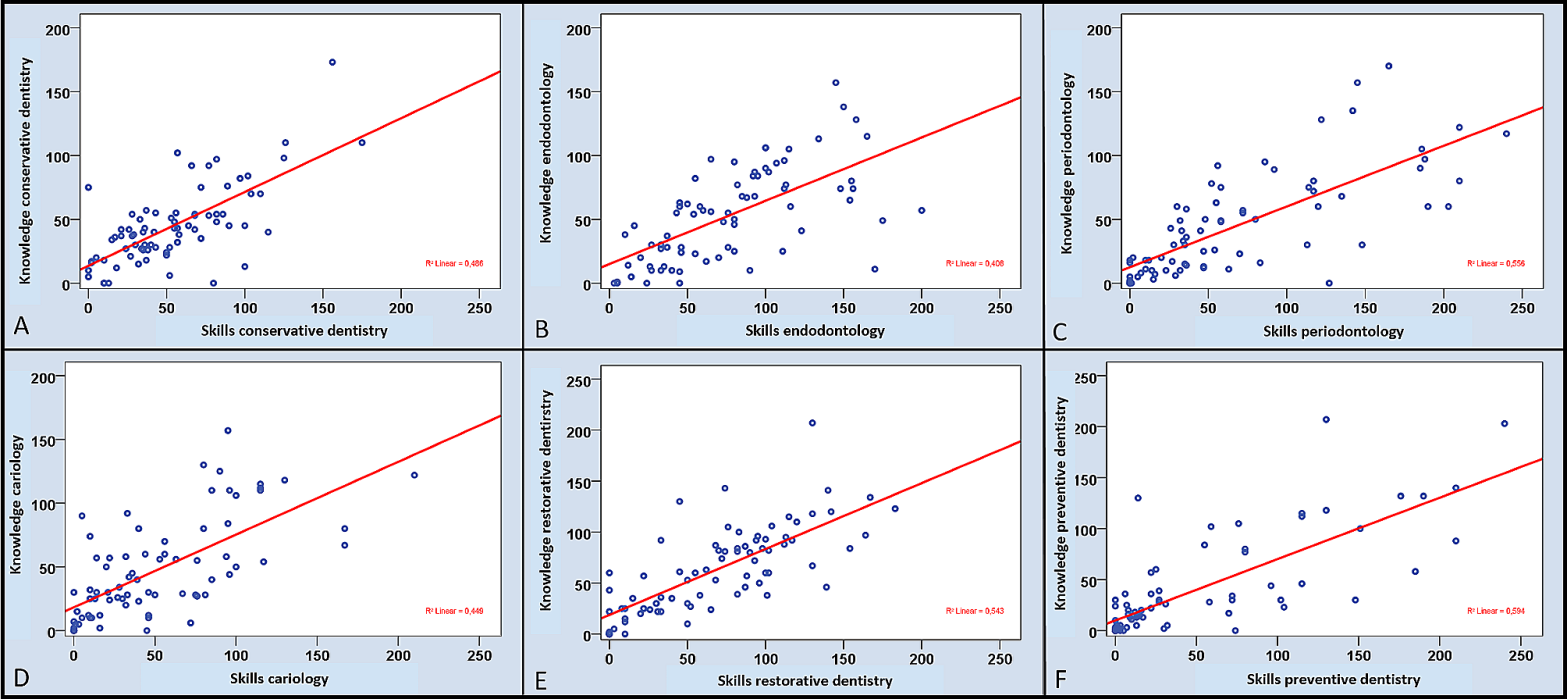
Results of the correlation analysis between self-perceived knowledge and skills in the total cohort (*n* = 75) for the whole field of conservative dentistry (**A**) and the five subdisciplines (**B**-**F**)

In the whole field of conservative dentistry and its sub-disciplines, significant correlations were evaluated between perceived theoretical knowledge and practical skills (*p* < 0.01). The strongest correlations were revealed for preventive dentistry (*r* = 0.802), followed by periodontology (*r* = 0.787), and restorative dentistry (*r* = 0.767). Overall, the positive correlations between perceived knowledge and skills were moderate or high (Table 4; Fig. 2).

**Table 4:** Correlations between knowledge, skills, and interests in the total cohort of 75 students

	Knowledge and skills		Interests and skills		Interests and knowledge	
	Spearman rho	*p* value	Spearman rho	*p* value	Spearman rho	*p* value
Whole field of conservative dentistry and periodontology	0.642	**< 0.01**	0.415	0.10	0.253	**0.03**
Endodontology	0.686	**< 0.01**	0.247	**0.03**	0.451	**< 0.01**
Periodontology	0.787	**< 0.01**	0.266	**0.02**	0.351	**< 0.01**
Cariology	0.685	**< 0.01**	0.144	0.22	0.366	**< 0.01**
Restorative dentistry	0.767	**< 0.01**	0.419	**< 0.01**	0.314	**0.01**
Preventive dentistry	0.802	**< 0.01**	0.126	0.28	0.240	**0.04**

Values are given as the distance between the object and subject disc in millimeters, shown as the mean values and standard deviation. Significant findings are expressed in bold font (*p* < 0.05)

### Correlations between perceived interests and skills

**Fig. 3:**
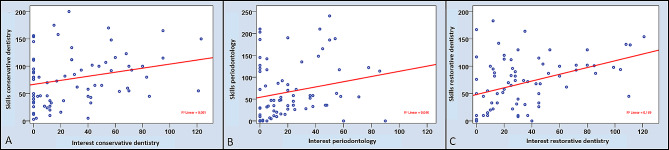
Results of the three significant correlation analyses between self-perceived skills and interests in the total cohort (*n* = 75) for the whole field of conservative dentistry (**A**) and periodontology (**B**) and restorative dentistry (**C**)

With regard to perceived interests and practical skills, only three significant correlations were revealed. A moderate positive correlation was found for restorative dentistry (*r* = 0.419, *p* < 0.01), while weak positive correlations were found for endodontology (*r* = 0.247, *p* = 0.03) and periodontology (*r* = 0.266, *p* = 0.02; Table 4; Fig. 3).

### Correlations between perceived interests and knowledge

**Fig. 4:**
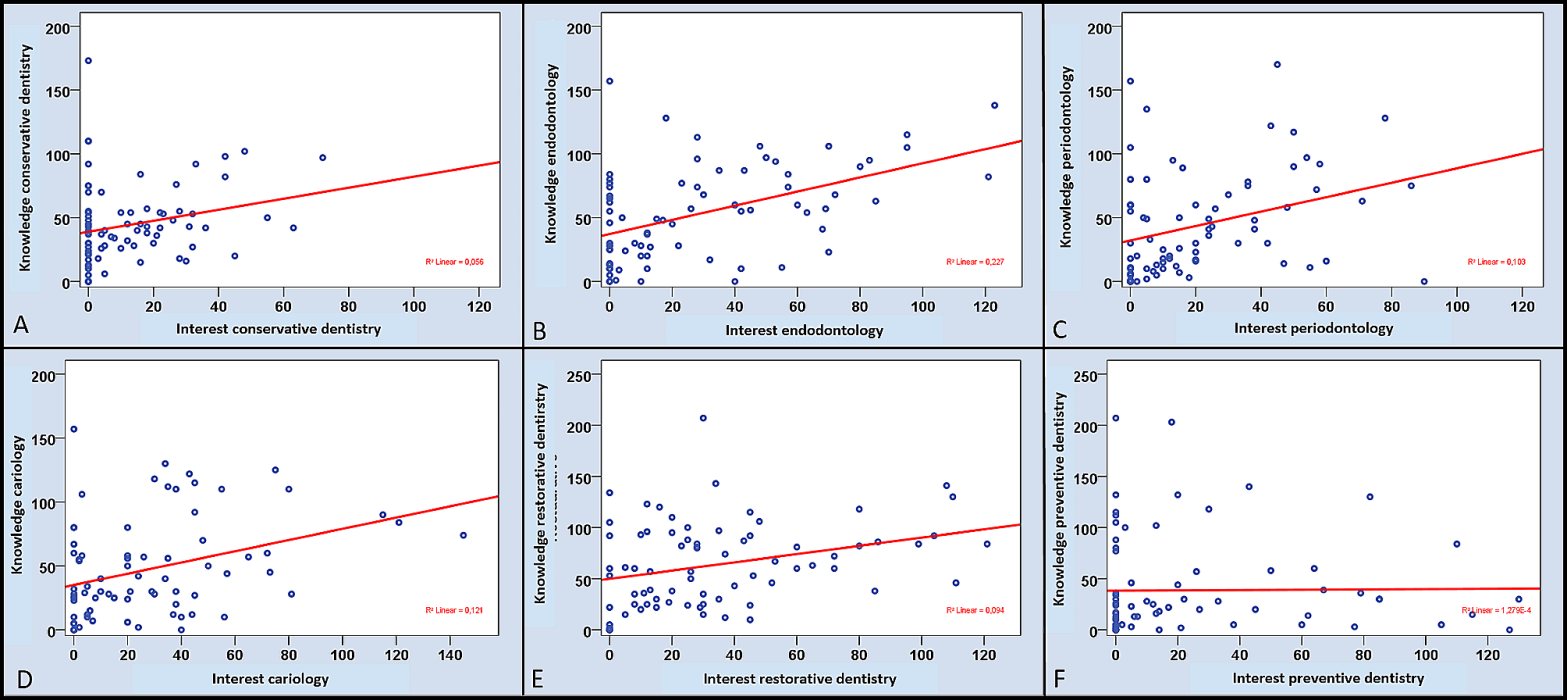
Results of the correlation analysis between self-perceived knowledge and interests in the total cohort (*n* = 75) for the whole field of conservative dentistry (**A**) and the five subdisciplines (**B**-**F**)

For the entire field of conservative dentistry and its five sub-disciplines, significant correlations were found with respect to perceived interests and theoretical knowledge (*p* < 0.05, Table 4). Therefore, the positive correlation for endodontology was moderate (*r* = 0.451, *p* < 0.01), but for all the other disciplines, the positive correlations were weak (*r* < 0.4, Table 4; Fig. 4).

## Discussion

### Main results

Overall, differences in subjectively perceived theoretical knowledge and practical skills in the overall field and sub-disciplines of restorative dentistry and periodontology were revealed in PRISM interviews between third-, fourth-, and fifth-year students. The PRISM-based interests in conservative dentistry and periodontology were high and comparable in the three groups. In the entire sample of 75 students, correlations were assessed between perceived knowledge, skills and interests. PRISM revealed that theoretical knowledge correlated with practical skills. Additionally, interests had a weak to moderate correlation with theoretical knowledge.

### Comparison with available literature

Although PRISM has been used in previous studies in the dental education context [8, 9], there are still limited data on this method in the context of teaching. In a previous study, PRISM was found to be reproducible and sensitive against changes in teaching and that there are significant differences in perceived knowledge and skills between third-, fourth-, and fifth-year dental students (*n* = 10 each) [8]. This leads to the hypothesis of this study that perceived knowledge and skills would increase with each additional year of dental studies. The present study confirmed this hypothesis for knowledge and skills but not for interests. Overall, the perception of greater knowledge and skills in students at higher years of study is not surprising. Previous studies showed that students in higher years of study perceive greater knowledge and/or skills, such as in orofacial pain [17], dental implants [18], or prosthodontics [19], than their colleagues with fewer years of study. Therefore, academic progress and experience appear to positively influence perceived competencies. This appears not only to be evident for perceived knowledge and skills but also for objectively measurable competencies. Different examinations confirm a measurable gain in knowledge and skills during dental studies [20, 21]. However, the results of the present study show that the field of endodontology appears to be an exception, as no differences were found regarding perceived knowledge and skills in this domain (see Tables 1 and 2). Based on the current monocentric study, it is unclear whether this is a local or generalizable phenomenon and an explanation remains speculative. It is worth noting that endodontics is regarded as highly challenging, where difficult cases are often referred to or managed by specialists in dental clinics, and this belief could decrease self-confidence and thus self-perceived competencies in this field. This would be in line with a previous survey-based study [22].

In the PRISM interviews, students’ interests in conservative dentistry and in its sub-disciplines were high, irrespective of their year of study. Students’ interests are quite relevant in dental education and postgraduate orientation, and several studies found that clinical experiences, such as clinical rotations and elective courses alongside mentoring programs, can nurture student interests [23–25]. Based on the findings of the current study, students in the field of conservative dentistry appear to be highly motivated, as their interest is consistently strong. In contrast to the methods in previous studies, which primarily used questionnaires, PRISM offers a new and promising visual approach to assess interests and could be used to illustrate interests in the different disciplines of dentistry to develop interest-based learning and teaching. This may be highly driven by students’ motivation. Previously, several authors have illustrated the potential benefits of individualized education and the importance of student motivation [26–28]. In this respect, PRISM-based assessment of interest and motivation may be a promising approach.

Another line of inquiry in the current study was the potential correlation between perceived knowledge and skills as well as the interests of the students. It was hypothesized that knowledge, skills, and interests would be correlated with each other. The results of the present study only partially confirm this hypothesis. First, knowledge and skills were reasonably correlated with each other. However, a previous study found a correlation between theoretical knowledge and practical skills in endodontics that was quite weak, with an *r* = 0.13 [15]. Another study, which was performed in the phantom course, also found a correlation between perceived competencies and results in practical exams (OSPE) [5]. This is in line with the PRISM-based results in the current study. Correlations between knowledge and skills, whether perceived or objectively assessable, appear plausible. Second, interests are correlated with theoretical knowledge but less so with practical skills. Practical skills are somewhat difficult to develop and require extensive practical training, which is often provided by simulation-based dental teaching events [29, 30]. Therefore, performance in practical skills may not be primarily interest-driven. However, theoretical knowledge may be influenced by students’ interests, which would support the use of PRISM to reveal interests as a basis for individualized education.

For clinical implementation, PRISM could be used for self-reflection of the students regarding their learning progress. Thereby, PRISM can be applied during a clinical course in dentistry for a continuous self-reflection of perceived learning progress [9]. Furthermore, it can be used during a preclinical course to reflect the development of skills during different modules or teaching events [8]. Overall, the students perceive a high value and benefit when using PRISM compared to a numerical scale [9]. This appears to be based on the personality of the method, its visual character and the fact that students perceive their perspective to be relevant for the teacher. In medical education, visual analogue scales (VAS) are repeatedly used in different situations [31, 32]. Basically, PRISM follows a similar approach, but has some relevant differences compared with a VAS. PRISM is haptic, whereby placing an object is a physical act, making it more operative. PRISM has no numeric scale but only a context, which limits the placement of the objects. Thus, PRISM cannot be transferred into a “good” or “correct” result, because it is individual and personally salient [7]. PRISM allows a completely personal form of reflection. This might be an important strength compared with a VAS. However, future research in the field should consider a comparison between PRISM and a VAS to depict a potential superiority of one method in dental education context.

### Strengths and limitations

The objectives of the current study are relevant to educational practice, and PRISM, a novel visual tool, was applied as an innovative approach. However, several limitations require consideration. First, this current study was a monocentric cross-sectional examination. This limits the generalizability of the results, leading to a pilot character of this cross-sectional study. In Germany, there are different settings of teaching conservative dentistry and its sub-disciplines. In addition, there are many differences in international settings of teaching those subjects. Thus, an international comparison of the differences would be needed to draw generalizable conclusions of using PRISM in this context. Moreover, a comparison of the subjective evaluation of the whole curriculum, especially including oral surgery, prosthodontics and orthodontics would be of interest and could be a valuable perspective for future research. To assess individual developments during dental education, a longitudinal assessment over the study time is planned. Second, the inclusion of volunteers, who may be more motivated or invested in dental educational issues, may somewhat bias the study cohort. The gender distribution is different between the study years in the current study, although this was not significant. The gender distribution of the three subgroups was influenced by the general gender ratio in the respective study year. Potentially, gender could be one influential factor of the PRISM task. Thus, current studies should recognize a potential gender effect. Given the limitations of the non-parametric analysis of the data in the current study, a statistical model using rows and columns simultaneously, possibly with gender as covariate, was not possible. Future study designs should consider this point as important analytical perspective. Additionally, the interviews being conducted by a teacher may result in both observational and interviewer bias. This may have influenced the way in which students self-assessed their knowledge and skills in the PRISM task. On the other hand, if PRISM is used in dental educational practice, the interviews would likely also be performed by their teacher. Thus, interviewer bias appears reasonable and close to the reality of teaching. However, in fact, the interviewer bias appears to relativize the findings in the current study, when the students reflect self-perceived relations between knowledge, skills and interests in PRISM. Therefore, it appears mandatory to use a neutral investigator in prospective studies to minimize the interviewer-bias. Because the measurement of distances was standardized, observational bias may be less relevant here. As described previously, PRISM requires some training and is sensitive to technique [8]. This limits the transferability of the study setting and findings. Future studies should consider this issue by developing manuals for the practical application of this method. Furthermore, it would have been of interest to investigate the relationship between PRISM and the objective knowledge and skills of the students. Essentially, the aim of using PRISM was to visualize the subjective competencies of students rather than teachers. This can be a basis for discussion, rather than an “assessment.” Therefore, investigating the relationship between PRISM and “objective” skills and knowledge may be of future interest but is not within the focus of the present study. In future projects, PRISM may be used alongside theoretical and/or practical exams (e.g., objective standardized clinical examinations) to evaluate this point. Overall, PRISM appears to be useful in the context of dental education. However, as a novel instrument, which may be confusing to interpret, it must be discussed critically. By nature, PRISM is a subjective tool that is only able to visualize an individual perspective. Any interpretation is highly context-dependent and must be made with caution. As mentioned above, visual self-evaluation using PRISM is more effective for students than for teachers and follows a highly student-centered approach. Growth and learning progress are always complex and individual, and while PRISM could be one supportive approach, it is not a complete solution. Future research projects should elaborate specific strategies to apply PRISM in teaching and illustrate potential fields of application for this novel visual instrument.

## Conclusion

PRISM can illustrate differences in perceived knowledge and skills in conservative dentistry and periodontology between third-, fourth-, and fifth-year undergraduate dental students. Moreover, correlations were found between perceived knowledge and skills, as well as between interests and knowledge. As a visual tool that can be used to illustrate the interests and learning needs of students, PRISM may be promising to support both students and teachers in dental education.

## Data Availability

The datasets used and/or analyzed during the current study are available from the corresponding author upon reasonable request. The data are not publicly available because of the pseudonymization and data protection guidelines according to ethics approval.

## Abbreviations

PRISM: Pictorial Representation of Illness and Self-Measure
